# Relationship between autistic traits and letter-recognition under attention to face-likeness: study using a henohenomoheji-type compound stimulus

**DOI:** 10.1038/s41598-023-46315-5

**Published:** 2023-11-03

**Authors:** Midori Sugiyama, Shinya Fujii, Masaki Mori

**Affiliations:** 1 Faculty of Policy Management, Keio University, 5322 Endo, Fujisawa, Kanagawa 252-0882 Japan; 2 Faculty of Environment and Information Studies, Keio University, 5322 Endo, Fujisawa, Kanagawa 252-0882 Japan

**Keywords:** Human behaviour, Psychology, Risk factors

## Abstract

This study aimed to clarify the relationship between autistic traits and letter information processing, specifically, the components of faces when attention is paid to face-like information. We created a new “henohenomoheji-type compound stimulus,” in which letters are placed in positions in such a way as to resemble a face. In Experiment 1, we examined the relationship between autistic traits and the participants’ performance in a letter-recognition task in which a henohenomoheji-type compound stimulus was used. The results showed a significant moderate negative correlation between Autism-Spectrum Quotient-Japanese Version (AQ-J) scores and letter-recognition sensitivity when the compound stimuli were arranged like a face. The letter-detection task was employed in Experiment 2 to examine how autistic traits affect tasks' performance with a lower cognitive load than in Experiment 1. We found no correlation between AQ-J scores and letter-detection sensitivity with or without face-like features. These results suggest that paying attention to faces reduces the participants’ performance in letter recognition, which represents a higher cognitive load in individuals with higher autistic traits. A major implication of this study is that the henohenomoheji-type compound stimuli can be applied to several cognitive tasks, such as cognitive processing in individuals with autistic traits.

## Introduction

Autism spectrum disorder (ASD) is a neurodevelopmental disorder characterized by deficits in individuals’ social communication and interactions, their repetitive patterns of behavior, and restricted interest^1,2^. Autistic traits can be assessed as a spectrum, regardless of diagnosis^3–7^. Several studies conducted among the general population and college students have reported associations between individuals’ autistic traits and their performance in cognitive tasks, such as the visuospatial working memory^8^, global information processing^9,10^, the perception of gaze direction^11^, the gaze cueing^12^, and the grouping effect on numericity estimation^13^. These acts of visuospatial processing are believed to be explained by the weak central coherence theory^14^, enhanced perceptual functioning model^15^, or the hypothesis on a specific zoom-out attentional impairment^16^. Since the relationship between autistic traits and visuospatial processing in the general population is inconsistent in the findings of previous studies^17–21^, accumulating evidence on visuospatial processing in the general population may lead to the understanding of the cognitive features of autistic traits.

Individuals with ASD are considered to have defects in recognizing faces owing to visuospatial processing characteristics^22,23^. The Difference between individual with ASD and typically-developing ones in face processing is well known in the studies that used behavioral^24–27^, neurological^25,28^, and eye-tracking^29–32^ measurements. One study has revealed that children with ASD make more errors in identifying faces with low-pass filters than they do faces with high-pass filters in face identification tasks^33^. Contrastingly, typically developing children make more errors on faces with high-pass filters than those with low-pass filters. Low-pass filters preserve the whole facial information, such as the arrangement of facial parts and impressions, and create a blurred image. High-pass filters highlight the importance of local information, such as facial contours and salient features. These findings suggest that individuals with ASD may process faces based on local, rather than, global information. However, spatial information processing for faces does not fully classify local features, such as eyes and mouth, and global features, such as the spatial arrangement of facial parts in the face, using spatial frequency methods.

One way to treat spatial arrangements of the face is to use face-like patterns, which is known as pareidolia phenomena^34–36^. Face-likeness is perceived from the spatial arrangement of eyes, nose, and mouth regardless of the facial parts themselves^37–39^. A previous study has examined whether individuals with ASD and typical development differ in the perception of face-likeness using a face-n-food task^40^. The study has found that individuals with ASD have higher perceptual thresholds than those with typical development for a face-like pattern created with food as in Giuseppe Archimboldo’s oil paintings as face-likeness. An electroencephalogram study has shown that individuals with ASD are less neural-responsive to face-like objects than typical development^41^. These results suggest that individuals with ASD have more difficulty perceiving face-likeness for objects that are not faces than individuals with typical development.

The finding that individuals encounter difficulty with face processing indicates a high cognitive load for face information processing. In general, it is known that attention to face-likeness increases detection sensitivity for stimuli that would induce pareidolia phenomena^42^. Studies have shown that individuals with ASD pay less attention to faces than typically developing individuals and pay more attention to faces and non-face objects alike^43,44^. A study investigating the interference of facial stimuli in an object detection task^45^ has found that typically developing individuals take longer to detect an object, such as a butterfly when a face is presented as a distractor. Contrastingly, the performance of individuals with ASD is not affected by the presence of a face as a distractor. These studies suggest that individuals with ASD have a higher cognitive load only when they pay attention to facial information. However, how autistic traits affect perceptual processing when they spontaneously attend to faces remains unclear.

This study aimed to investigate the effect of individuals’ autistic traits on their letter information processing while paying attention to face-likeness information. We created a new stimulus called the henohenomoheji-type compound stimulus, inspired by Navon’s hierarchical letter stimuli (e.g., a large “H” composed of multiple small “S”)^46^ and Japanese calligraphy (Henohenomoheji). Henohenomoheji, known for an old Japanese drawing song^47^ and used in the opening ceremony of the Tokyo Olympics in 2020^48^, is spatially arranged like a face using letters. Therefore, the henohenomoheji-type compound stimuli can be used for dual-task: letter-detection tasks like Navon’s hierarchical letter stimuli and face-likeness detection tasks like the pareidolia phenomenon. Face-likeness would be perceived through the global processing of the spatial arrangement. According to the weak central coherence theory and the hypothesis on a specific zoom-out attentional impairment, the more autistic traits individuals have, the more load on global processing and the less load on the local information recognition, despite the degree of face-likeness. Since individuals with ASD are considered to have difficulty with face recognition, we expect stimuli with high face-likeness to be an extraneous cognitive load on other information recognition for those with higher autistic traits.

## Experiment 1

### Methods

#### Ethical declaration

All procedures of this study were performed in accordance with the “Declaration of Helsinki regarding medical research involving human subjects.” This study was approved by the Research Ethics Committee of Keio University SFC on May 19, 2021, with approval number 341. The experiment was conducted between August 2021 and September 2023. Written informed consent was obtained from all participants prior to their participation.

#### Participants

Seventy-one undergraduate students, graduate students, and researchers without experience in the research topic participated in this study (35 females, 36 males). The participants’ mean age was 21.96 years ($SD=4.32$), and none of them reported any visual dysfunctions. The sample size was determined before the beginning of the experiment concerning previous studies^17,49^. A post-hoc power analysis for one-tailed correlation analysis was conducted by G*Power 3.1.9.7^50,51^. The results showed that $1-\beta$ exceeded .80 when $\alpha$ was 0.05, the effect size was 0.30, and the sample size was 71.

#### Apparatus

Visual stimuli were presented on a monitor (BenQ, ZOWIE XL2546S, 24.5 in., a screen resolution $1920\times 1080$ pixels) with a 60 Hz refresh rate in a darkroom. The visual distance was set at 47.5 cm by using a chin rest. The participants responded by using a response pad (Cedrus Corp., RB-540 Response Pad). PsychoPy^52,53^ was used to create the experimental program. We conducted the experiment with a computer (Dell Technologies, XPS13 9350).

#### Stimuli

**Figure 1:**
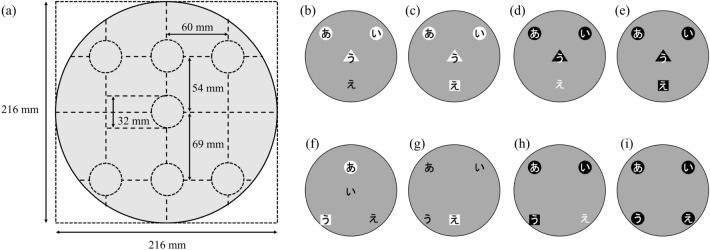
Henohenomoheji-type compound stimuli. (**a**) Construction of the stimulus. (**b**–**e**) Examples of high face-like stimuli. (**f**–**i**) Examples of low face-like stimuli.

We created stimuli called henohenomoheji-type compound stimuli during this study. Figure 1a shows a diagram of the construction, the size, and the arrangement of the stimuli. The stimuli comprised Japanese letters called hiragana; four letters were placed in four of the seven spots within a $26.5^\circ$ diameter circle with the visual range set at 47.5 cm. In some conditions, three shapes (circle, triangle, or square) were placed behind the letters. Letters in henohenomoheji-type compound stimuli were either black or white. Shapes behind the letters were either white or black, which was always different from the color of the letters. Each spot where the letters and shapes were placed was $3.9^\circ$. Fifteen regularly arranged stimuli were prepared. To define the degree of face-likeness, we conducted a preliminary evaluation experiment. Twenty-one participants were asked to rate face-likeness from 1 (low) to 7 (high). These data were analyzed using a non-hierarchical cluster analysis of the *k*-means method. As a result, the stimuli were classified into two optimal clusters using the highest silhouette coefficients and maximum gap for all clusters. The first cluster consisted of nine stimuli (e.g., Fig. 1b–e). The second cluster consisted of six stimuli (e.g., Fig. 1f–i). The face-likeness score of the first cluster ($M=5.357, SD=1.488$) was significantly higher than the face-likeness score of the second cluster ($M=1.992, SD=1.400$) based on a *t*-test ($t(125)=-19.172, p <0.001, \mathrm {Cohen's}~d = 0.201$). The first cluster was defined as the high face-likeness group and the second cluster as the low face-likeness group. Irregularly arranged stimuli consisted of four letters (hiragana) and three types of shapes. The letters and shapes on the irregularly arranged stimuli were randomly placed in seven spots like those on the regularly arranged stimuli.

#### Psychometric questionnaire

Autistic traits were assessed through Autism-Spectrum Quotient-Japanese Version (AQ-J)^54,55^. The AQ-J is developed based on the original version of Autism-Spectrum Quotient (AQ)^3^, which is a self-administrated questionnaire used to assess the extent of a wide range of autistic traits regardless of whether a person is diagnosed with ASD^7^. AQ has been shown to have high retest reliability among college students^56^. AQ has also been translated into various languages, such as Chinese^57,58^, French^59,60^, Bahasa Malaysia^61^, German^62^, Italian^63^, Turkish^64^, Polish^65^, and Hebrew^66^. Participants responded to 50 items on a four-point Likert scale (“strongly agree,” “slightly agree,” “slightly disagree,” and “strongly disagree”). We converted those responses to 1 for the presence of autistic traits and 0 for the absence of autistic traits after considering whether the item was reversed. The probability of individuals with ASD scoring higher than 33 on the AQ-J is shown to be more than 90% in a Japanese sample^54,55^. Conversely, less than 3% of typically developing individuals score higher than 33. The AQ-J has been previously assessed as having high test-retest reliability and moderate-to-high internal consistency.

#### Procedure

**Figure 2:**
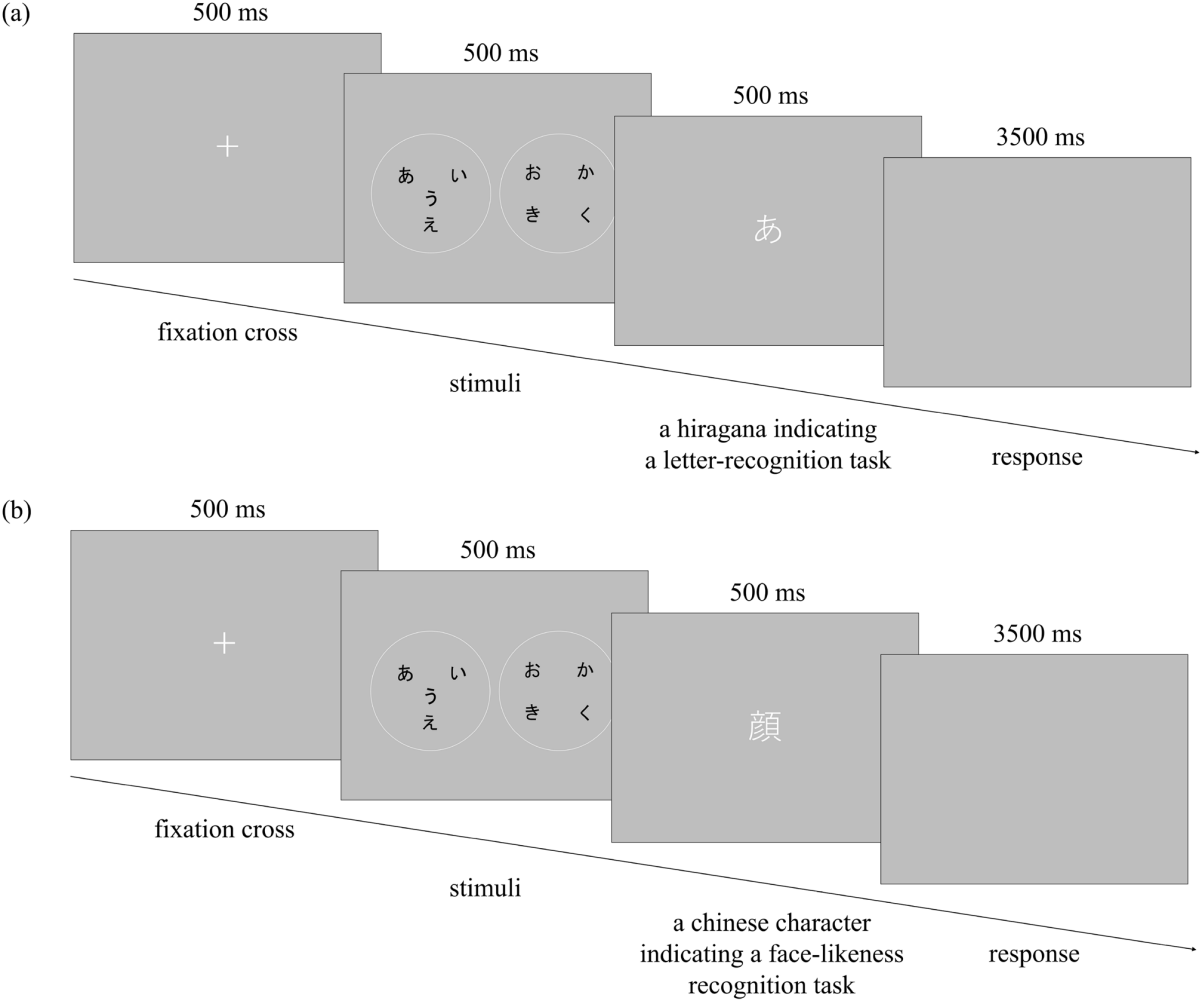
Schematic diagram of trials. (**a**) Letter-recognition task, (**b**) Face-likeness recognition task.

Figure 2 illustrates procedures followed in the letter-recognition task (Fig. 2a) and a face-likeness recognition task (Fig. 2b). A fixation cross was presented for 500 ms, followed by the two experimental stimuli for 500 ms. A letter was then presented, which was either a hiragana indicating a letter-recognition task, or a kanji (Chinese character) representing a face, indicating a face-likeness recognition task. In the letter-recognition task, the participants were instructed to press the left key when a target was present and the right key when a target was absent. In the face-likeness recognition task, the participants were instructed to press the left key when a stimulus looked like a face and the right key when none of the stimuli looked like a face. Regardless of the letter color on the stimuli, a target letter was always written in white. The maximum response time was 3500 ms and a subsequent trial was initiated. Stimuli used for both letter-recognition and face-likeness recognition tasks were always drawn from the 15 regularly arranged stimuli in Fig. 1b–i and one irregularly arranged stimulus, as in Fig. 1f–i. The experiment consisted of 120 trials of letter-recognition tasks (15 regularly arranged stimuli $\times$ 8 repetitions) and 24 trials of face-likeness recognition tasks. The order of the tasks was randomized in the experiment. Participants had to focus on both letters on the stimuli and the face-likeness of the stimuli. A target letter was present in 60 out of the 120 trials. The location of the target letter was counterbalanced across the 120 trials. The left and right positions of the experimental stimuli were randomly presented. The collected data were analyzed using the statistical software of R version 4.1.0^67^ and R Studio version 1.4.1717^68^. The “psycho” package in R^69^ was used to calculate sensitivity ($d'$) and bias (*c*) indices for each participant. The “psych” package in R^70^ was used for the calculation of Cronbach’s alpha and McDonald’s omega. The “NbClust,” “cluster,” and “factoextra” packages in R^71–73^ were used for cluster analysis.

#### Data processing criteria

High face-like stimuli (the top 6 types in the high face-likeness cluster out of 9 types) and low face-like stimuli (6 types in the low face-likeness cluster) were included in the analysis. The total of the trials was 96 (12 types $\times$ 8 repetitions). Twenty-four trials (the worst 3 types in the high face-likeness cluster out of 9 types) were excluded from the analysis. Performances on two types of tasks were evaluated by a signal detection theory, which uses hits and false alarms to compute the indices of sensitivity ($d'$) and bias (*c*). The indices were calculated separately for the high face-likeness condition, low face-likeness condition, and combined condition (containing both high and low face-like conditions). In trials where the target was present, the response was classified as “hit” if the response was “present” and “miss” if the response was “absent.” In trials where the target was not present, the response was classified as “correct rejection” if the response was “absent” and “false alarm” if it was “present.” The total number of responses in which the participants judged the stimuli to be a face was calculated for the face-likeness recognition task.

The data from 64 participants were used in the analysis. The data from 7 participants were excluded based on the acceptance criteria: (a) error during data collection due to an issue of equipment connection ($n=1$) and (b) data for participants with the outliers identified by the 2 SD method for sensitivity or bias in each condition ($n=6$).

### Results

#### AQ-J

The scores on AQ-J ranged from 4 to 39 ($M = 21.03, SD = 8.61$). Nine participants scored above the cutoff value of 33 on AQ-J. The normality of the data was confirmed using the Jarque-Bera test ($JB = 2.732, p =0.255$). Internal consistency reliability of the AQ-J for the data in this study was assessed to be moderate to high by Cronbach’s alpha and McDonald’s omega coefficients ($\alpha =0.82, \omega =0.75$).

#### Letter-recognition task

To check whether the participants could recognize the presence or absence of the target letter above the chance level, a one-sample *t*-test (5% level of significance) was used to ascertain whether the $d'$ was greater than 0. Consequently, despite the degree of face-likeness, $d'$ was significantly higher than the chance (high face-likeness condition: $t(63) = 10.503, p < 0.001, M = 0.473, SD = 0.360, \mathrm {Cohen's}~d = 1.313$; low face-likeness condition: $t(63) = 10.277, p < 0.001, M = 0.454, SD = 0.353, \mathrm {Cohen's}~d = 1.285$). The result suggests that despite the face-likeness of the stimuli, the participants could correctly determine the presence or absence of a target letter at a rate higher than if it were accidental.

A paired *t*-test comparing the performance on the letter-recognition task in high face-likeness and low face-likeness conditions was conducted to assess the effect of face-likeness (5% significance level). No statistical difference was found between the performance on the letter-recognition task in the face-likeness conditions ($t(63) = 0.332, p = 0.741, \mathrm {Cohen's}~d = 0.007$). Therefore, a degree of face-likeness did not affect the letter-recognition task.

The relationship between autistic traits and participants’ performance in letter-recognition tasks across high and low face-likeness conditions was analyzed. Correlation analysis showed no significant correlation between AQ-J scores and $d'$ value ($r = -0.046, p =0.717$). The results indicate that overall letter-recognition performance is not related to autistic traits. Response bias was also not related to autistic traits ($r=0.027, p=0.833$).

**Figure 3:**
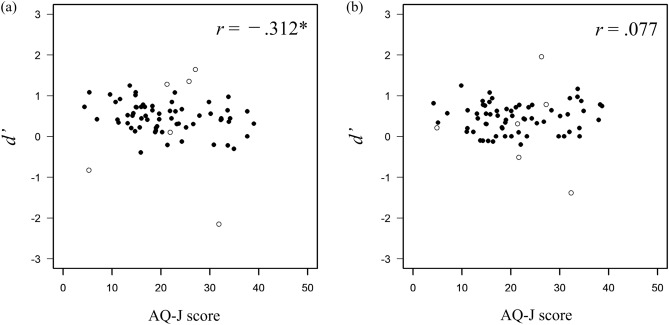
Relationship between the AQ-J score and sensitivity for letter recognition in (**a**) the high face-likeness condition and (**b**) the low face-likeness condition. The asterisk indicates $p<0.05$. Filled circles indicate data used in the analysis and open circles indicate data excluded from the analysis.

**Figure 4:**
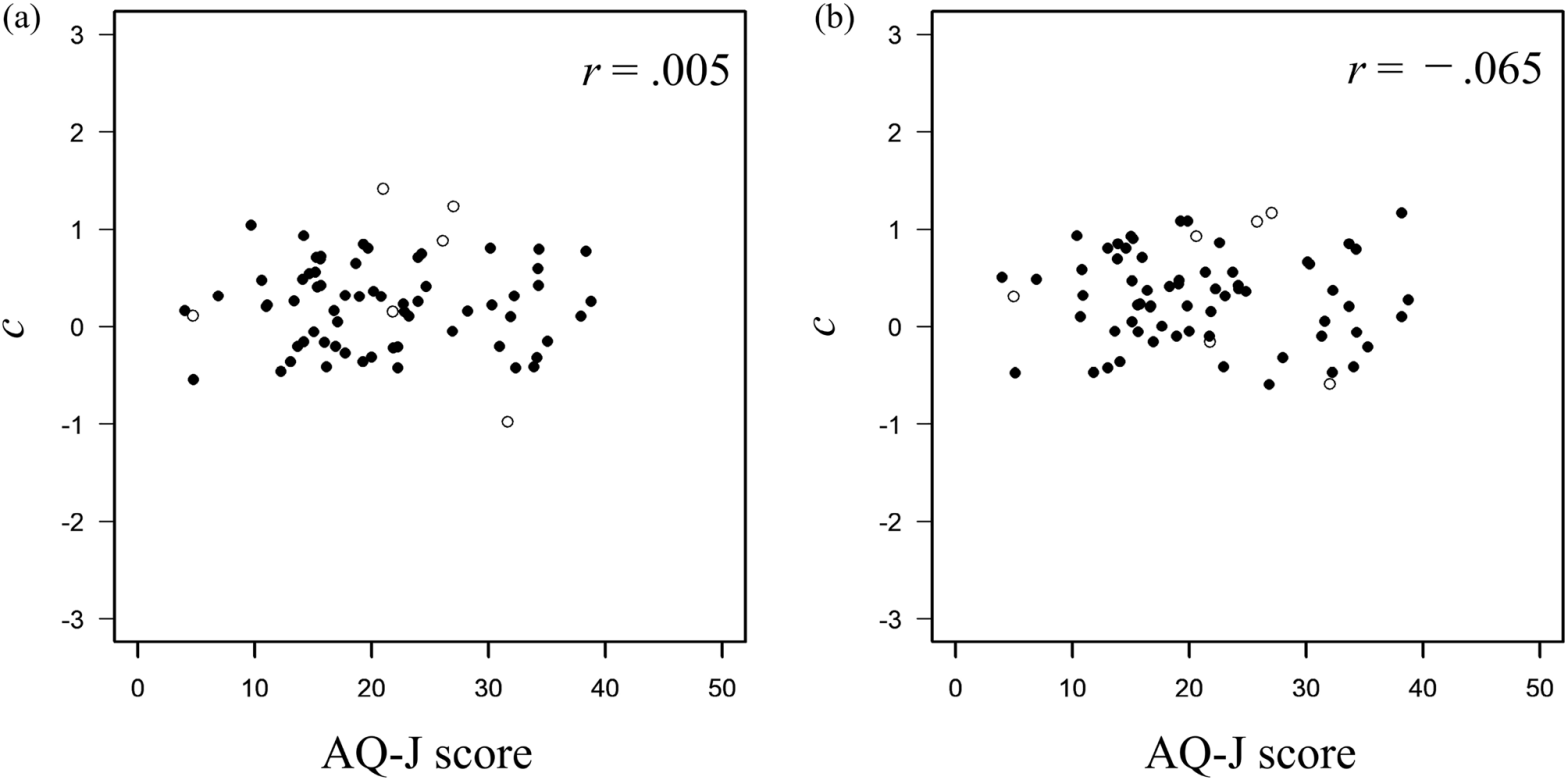
Relationship between the AQ-J score and response bias for letter recognition in (**a**) the high face-likeness condition and (**b**) the low face-likeness condition. Filled circles indicate data used in the analysis and open circles indicate data excluded from the analysis.

Regarding the relationship between autistic traits and local information recognition ability, correlations were calculated across the AQ-J scores and $d'$ in each face-likeness condition. A correlation analysis was also conducted (5% significance level). Figure 3a shows the relationship between the score of AQ-J and the performance on the letter-recognition task in the high face-likeness condition, and Fig. 3b shows the relationship in the low face-likeness condition. In the high face-likeness condition, the results showed a significant moderate negative correlation between the AQ-J score and $d'$ on letter-recognition task ($r = -0.312, p =0.012$). In the low face-likeness condition, no significant correlation was found between them ($r =0.077, p =0.546$). These results suggest that when face-like stimuli are presented, the more autistic traits the participants have, the worse their performance on letter-recognition tasks. Conversely, autistic traits did not affect the participants’ performance in letter-recognition tasks when non-face-like stimuli were presented. Response biases of the letter-recognition task were examined. Figure 4a shows the relationship between the AQ-J score and response bias of the letter-recognition task in the high face-likeness condition, and Fig. 4b shows the relationship between them in the low face-likeness condition. There was no significant relationship between the scores of AQ-J and *c* in both conditions (high face-likeness condition: $r=-0.005, p=0.968$, low face-likeness condition: $r=-0.065, p=0.609$).

#### Face-likeness recognition task

To assess the effect of autistic traits on face-likeness recognition, the correlation between the number of responses in the face-recognition task and AQ-J was calculated. A correlation analysis was also conducted (5% significance level). The result showed no significant correlation between them ($r=-0.094, p=0.463$). Thus, autistic traits did not affect face-likeness recognition.

### Discussion

Experiment 1 aimed to reveal whether the effect of face-like global information that places a load on local information recognition differs depending on autistic traits. Henohenomoheji-type compound stimuli were used for the letter-recognition task. Performance for letter-recognition tasks in high and low face-likeness conditions was measured using $d'$. In the high face-likeness condition, letter-recognition sensitivity decreased with the increase of autistic traits in the participants. In contrast, no correlation was found between autistic traits and letter-recognition sensitivity in the low face-likeness condition. Since the AQ-J scores and the number of responses in the face-recognition task were not related, the participants’ ability to judge henohenomoheji-type stimuli as a face was not associated with autistic traits. Therefore, in a letter-recognition task using compound stimuli, face-likeness as global information interferes with local information recognition in individuals with higher autistic traits. Furthermore, response biases on the letter-recognition task were not associated with autistic traits in high and low face-likeness conditions. This result can be regarded as not being caused by a response bias in letter recognition.

In Experiment 1, the target that indicated whether the task was a letter-recognition task or a face-likeness recognition task appeared after the stimuli were presented. Therefore, it is necessary to hold both tests in the working memory at the point when the stimuli are displayed. In other words, Experiment 1 was a cognitively demanding task in which participants engaged in a dual task of letter recognition and face-likeness recognition. To examine this possibility, whether the lowering of the cognitive load would produce different results should be determined. To investigate whether attention to face-likeness affects individuals’ performance in a task only when the cognitive load is high, we conducted a controlled psychological experiment (letter-recognition task) with a low cognitive load using the same stimuli and similar experimental paradigm as in Experiment 1.

## Experiment 2

### Purpose

Experiment 2 aimed to examine whether the presence or absence of face-like information in the stimuli makes a difference in the association between individuals’ autistic traits and their ability to detect local information when the cognitive load is lighter than it was in Experiment 1.

### Methods

#### Ethical seclaration

Ethical considerations in Experiment 2 were the same as in Experiment 1.

#### Participants

Seventy-two undergraduate students, graduate students, and researchers without prior experience in this research topic participated in this study (36 females, 36 males). The mean age participants was 21.93 years ($SD=4.30$), and mean AQ-J score was 20.78 ($SD=8.74$). Seventy-one participants were the same as in Experiment 1.

#### Apparatus, stimuli, and psychometric questionnaire

The apparatus, stimuli, and psychometric questionnaire used in Experiment 2 were the same as in Experiment 1.

#### Procedure

**Figure 5:**
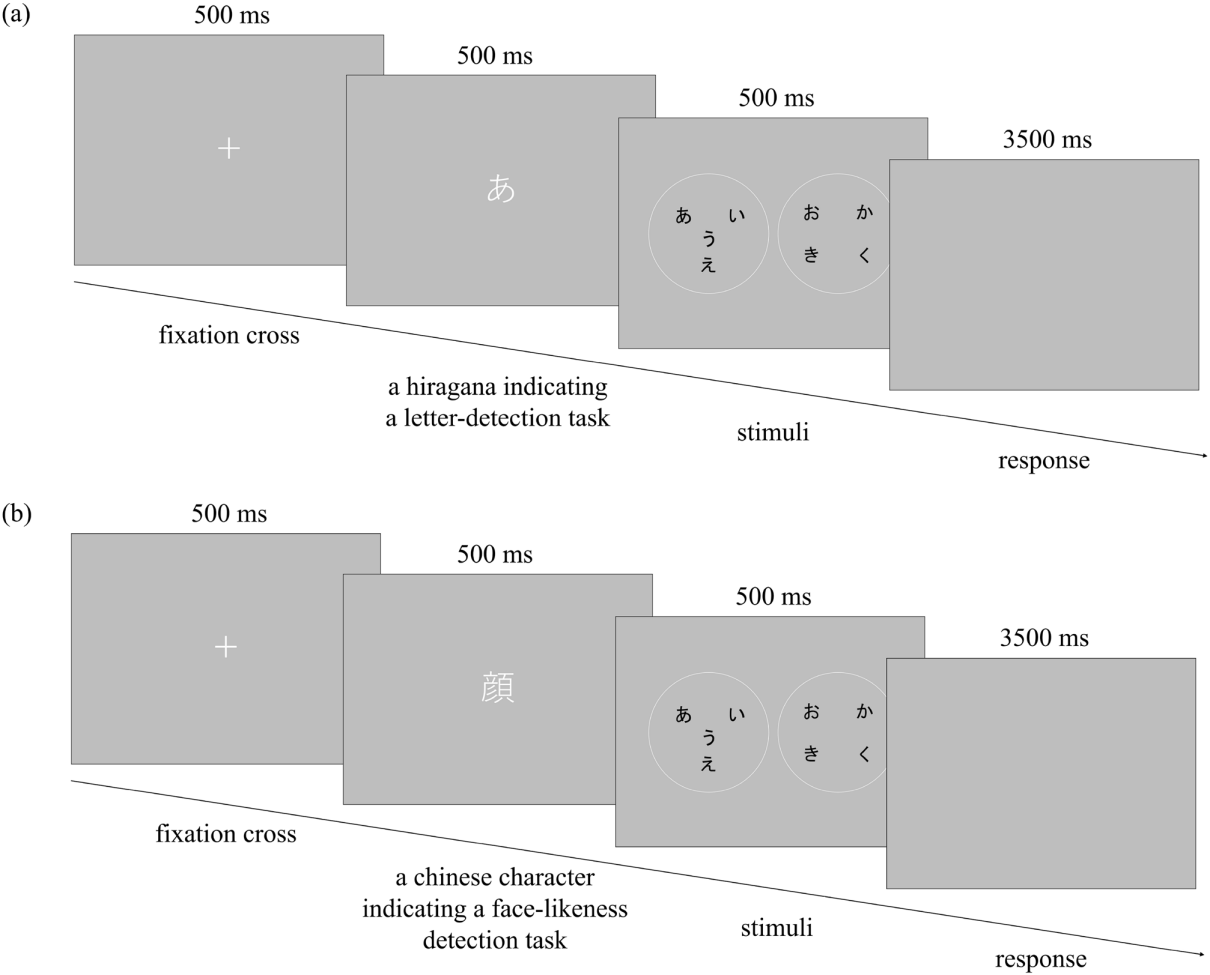
Schematic diagram of trials. (**a**) Letter-detection task, (**b**) Face-likeness detection task.

The experimental procedure was the same as in Experiment 1, except for the following points. In Experiment 2, participants were presented with either a single hiragana letter (for the letter-detection task; Fig. 5a) or a kanji, a character which means “face” (for the face-likeness detection task; 5b), as a target before observing the experimental stimuli. This procedure allowed the target to be known prior to displaying the stimuli.

#### Data processing criteria

The criteria of face-like stimuli in Experiment 2 were the same as in Experiment 1. The data from fifty-nine participants were used in the analysis. The data from 13 participants were excluded based on the same acceptance criteria in Experiment 1 (a: $n=2$, b: $n=11$).

### Results

#### Letter-detection task

To assess whether $d'$ was above the chance level of 0, a one-sample *t*-test was conducted (5% significance level). The results showed that regardless of the degree of face-likeness present or absent in the experimental stimuli, $d'$ was significantly higher than chance level (high face-likeness condition: $t(58) = 25.584, p < 0.001, M = 1.400, SD = 0.416, \mathrm {Cohen's}~d = 3.366$; low face-likeness condition: $t(58) = 22.980, p < 0.001, M = 1.216, SD = 0.407, \mathrm {Cohen's}~d = 2.992$).

The participants’ performance in the letter-recognition task in the high and low face-likeness conditions was compared to assess the effect of face-likeness in general. A paired *t*-test revealed a significant difference between the $d'$ for the letter-detection task under the two conditions ($t(58) = 2.638, p = 0.011, \mathrm {Cohen's}~d =0.057$). The results indicated that the performance of letter detection is better in the high face-likeness condition than in the low face-likeness condition.

The relationship between autistic traits and the participants’ performance in letter-detection tasks across high and low face-likeness conditions was examined. Correlation analysis revealed no significant correlation between AQ-J scores and $d'$ ($r =0.085, p =0.522$). Therefore, the participants’ overall letter-detection performance was not related to the number of autistic traits. The AQ-J score was also unrelated to *c* on the letter-detection task ($r=-0.014, p =0.914$).

**Figure 6:**
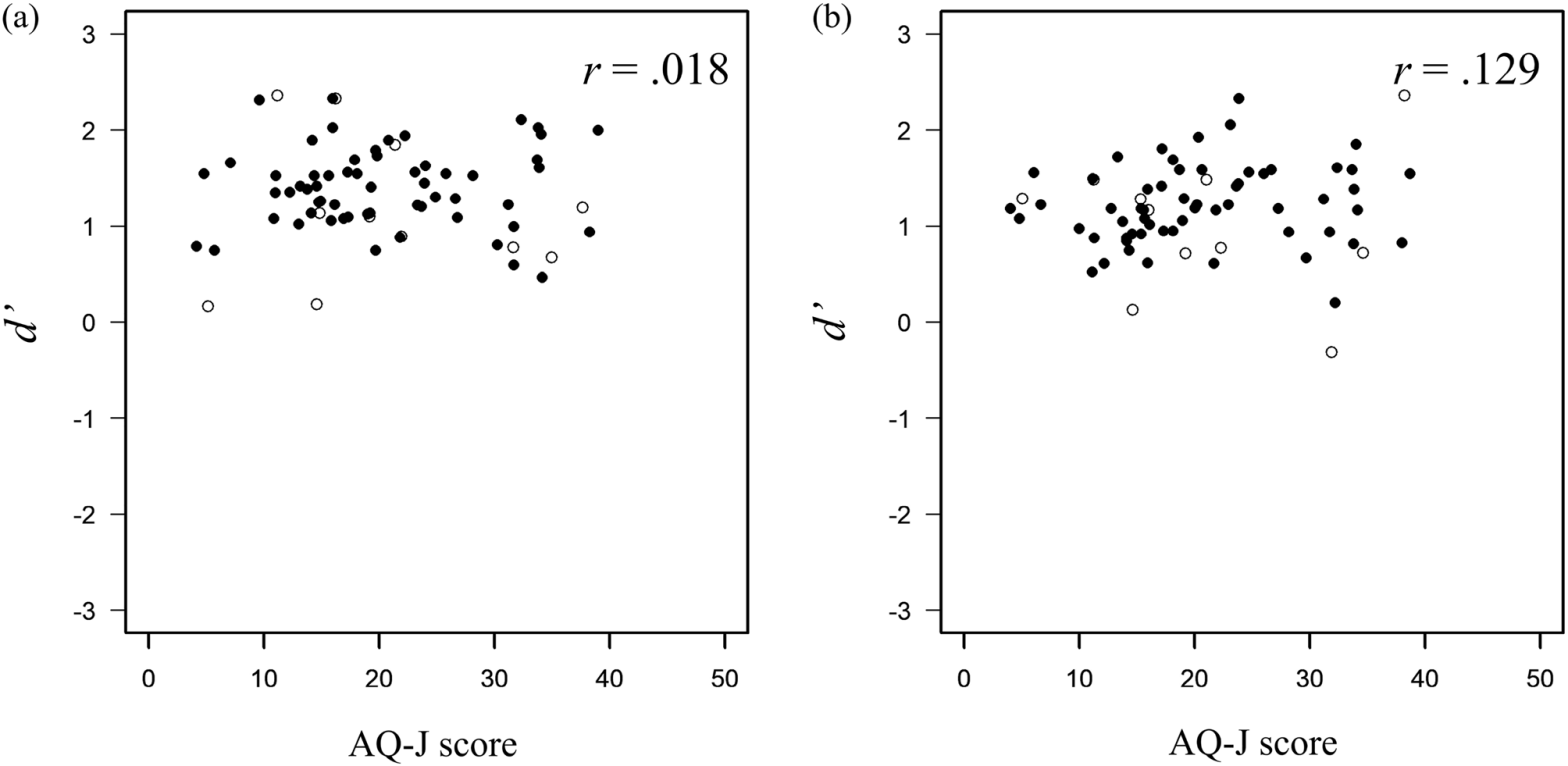
Relationship between AQ-J scores and sensitivity for letter-detection in (**a**) high face-likeness condition and (**b**) low face-likeness condition. Filled circles indicate data used in the analysis and open circles indicate data excluded from the analysis.

**Figure 7:**
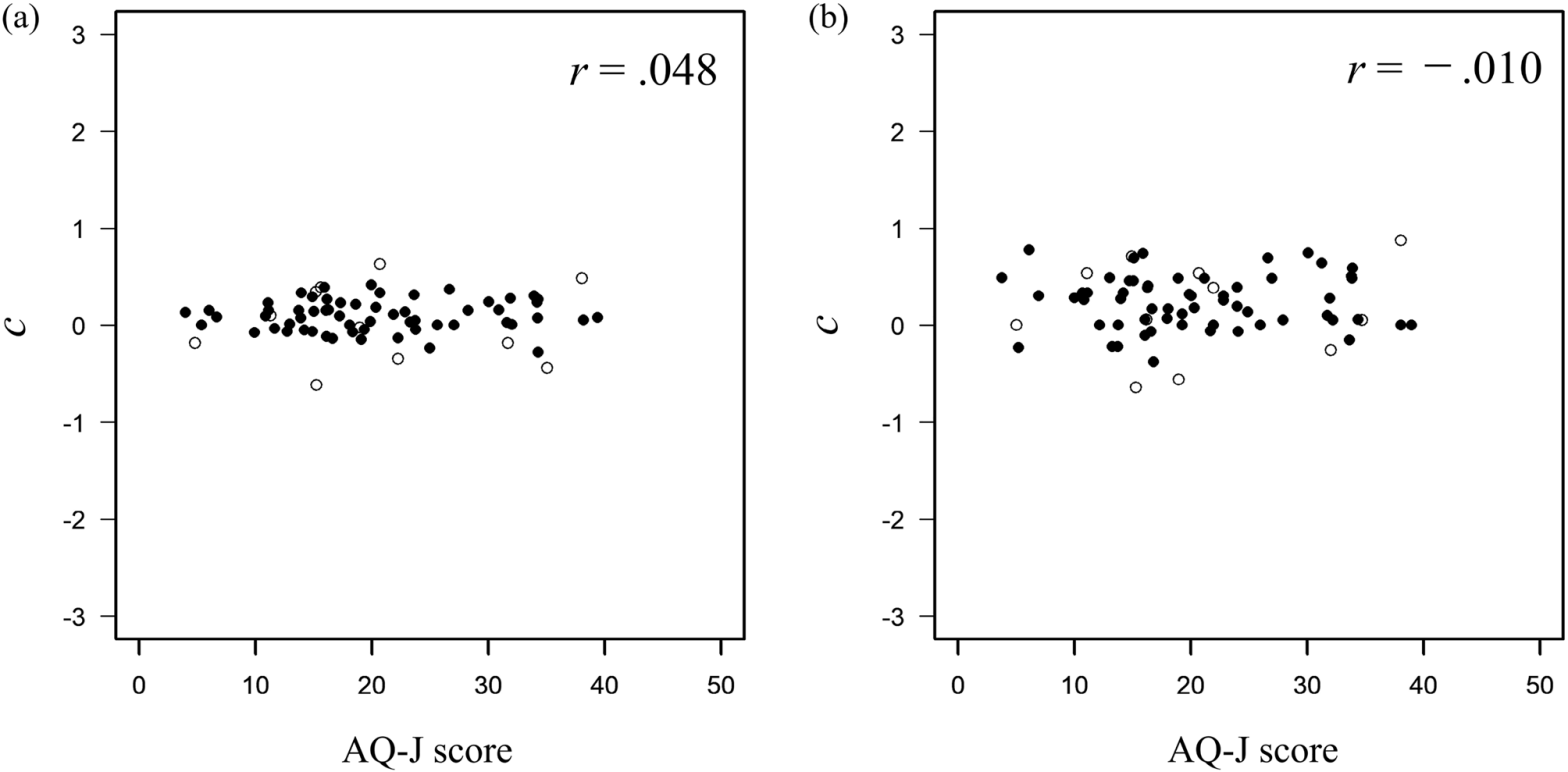
Relationship between AQ-J scores and response bias for letter-detection in (**a**) high face-likeness condition and (**b**) low face-likeness condition. Filled circles indicate data used in the analysis and open circles indicate data excluded from the analysis.

To determine whether the effect of the face-likeness of the stimuli on a letter-detection task depends on autistic traits, the correlations between AQ-J scores and $d'$ in each face-likeness condition were analyzed. Correlation analysis was also conducted. In both conditions, the results indicated that $d'$ for the letter-detection task and the AQ-J scores was not related (high face-likeness condition: $r = 0.018, p = 0.891$, Fig. 6a; low face-likeness condition: $r =0.129, p =0.330$, Fig. 6b). Furthermore, the performance on the letter-detection task was not related to autistic traits despite the presence of face-like stimuli. There was no correlation between autistic traits and response bias in the letter-detection tasks under high or low face-likeness conditions. The correlation analysis found no significant correlations between AQ-J scores and *c* (high face-likeness condition: $r=0.048, p=0.717$, Fig. 7a; low face-likeness condition: $r=-0.010, p=0.941$, Fig. 7b).

#### Face-likeness detection task

To evaluate the influence of autistic traits on face-likeness detection, we calculated the correlation between the number of responses in the face-detection task and the AQ-J score. A correlation analysis was also performed, which showed no significant correlation ($r=-0.024, p=0.854$). Therefore, autistic traits did not affect face-likeness recognition.

### Discussion

In Experiment 2, we aimed to determine whether face-like arrangements affect local information detection, which required lower cognitive load. In contrast to Experiment 1, autistic traits were not related to performance on the letter-detection task in either high or low face-likeness conditions (high face-like stimuli: $r=0.020, p=0.881$, low face-like stimuli: $r=0.050, p=0.706$). Response biases in the letter-recognition task were also not associated with autistic traits in either condition. Furthermore, $d'$ was higher in Experiment 2 than in Experiment 1 in both conditions (high face-likeness condition: $\Delta d'=0.927$; low face-likeness condition: $\Delta d'=0.762$). These results suggest that the task was less difficult and less cognitively demanding in Experiment 2 than in Experiment 1. We concluded that the appearance of a face does not suggest that the performance of letter information detection by individuals with higher and lower autistic traits is not inhibited when the cognitive load is low.

## General discussion

This study aimed to investigate whether the effect of face-like global information elicited during the performance of local information-recognition and detection tasks differs depending on autistic traits by using henohenomoheji-type compound stimuli. We assessed the relationship between the autistic traits in the general population and the letter-recognition task in both conditions, wherein face-like stimuli were present and absent. In Experiment 1, the participants’ performance in the local information-recognition task decreased with the increase of autistic traits in the high face-likeness condition ($r=-0.312, p=0.012$). However, no correlation was found between autistic traits and task performance in the low face-likeness condition ($r=0.077, p=0.546$). In Experiment 2, the letter-detection task was conducted to determine whether the correlation between autistic traits and the effect of face-like global arrangements would differ if the cognitive load of a task was lower than it was in Experiment 1. T﻿he results showed no relationship between autistic traits and the participants’ performance in letter-detection tasks despite the presence of face-like information (high face-like stimuli:$r=0.020, p=0.881$, low face-like stimuli:$r=0.128, p=0.329$). These results indicate that only in a dual task with high cognitive load using compound stimuli does face-likeness as global information interfere with local recognition in individuals with higher autistic traits.

The present study suggests that global face likeness arrangements may inhibit the tasks relevant to local information processing, requiring high cognitive load in individuals with more autistic traits. This hypothesis implies that the more autistic traits an individual has, the more difficulty they face in processing face-likeness global information. Therefore, the weak central coherence theory or the hypothesis on a specific zoom-out attention impairment cannot fully explain this outcome. These findings differ from those of a previous study^45^, which reports that faces do not become distractors in individuals with ASD. In the previous study, the participants are not asked to focus on facial images while detecting a target in a visual search task. Therefore, face-likeness may have played a role as a distractor during high cognitive load in the present study, especially in participants with higher autistic traits.

The relationship between autistic traits and the individuals’ performance in letter-recognition tasks depends on a degree of face-likeness as global information to an extent. Performance in the local information-recognition task decreased with the higher autistic traits in the high face-likeness condition. Conversely, no correlation was found between autistic traits and task performance on recognition tasks in the low face-likeness condition. These results suggest that autistic traits are not related to working memory in the task employed in this study. Previous studies have reported that the effect of autistic traits on working memory depends on the tasks^74–76^. A prior study on weak central coherence theory explains difficulties in visuospatial working memory and face recognition in individuals with ASD^75^. However, there are several differences between the previous studies and this study. The previous study uses a letter-recognition task, which has not been used as a working memory task in individuals with autism or high autistic traits. Moreover, the present study reveals that the absence or presence of face-like global stimuli is important for the correlation between autistic traits and letter-recognition tasks. Further experiments are required to identify models and hypotheses that explain the results of this study.

The new stimulus, the henohenomoheji-type compound stimulus, can be used for recognition and detection tasks. In henohenomoheji-type compound stimuli, faces are arranged as global information, and letters are arranged as local information. Hierarchical compound stimuli typically comprise geometric shapes or letters^18,34,46^. Previous studies that employed face recognition tasks used face stimuli. Therefore, global and local information could not be separated, or the local component conveyed by face-like arrangements was meaningless. Henohenomoheji-type compound stimuli are unique because they provide both facial information as social stimuli and meaningful letters. These characteristics make henohenomoheji-type compound stimuli useful for several cognitive tasks, such as understanding the cognition of individuals with ASD, who convey abnormal local and global information processing.

The present study had some limitations. First, henohenomoheji-type compound stimuli cannot identify whether global information is processed as faces or objects. To perceive face-likeness from a henohenomoheji-type compound stimulus, the stimulus must be processed globally. Further studies using compound stimuli other than faces, such as cars and houses, are required to compare objects and face processing. Second, participants were general undergraduate, graduate students, and researchers. Therefore, the results might have differed if the experiment included individuals diagnosed with ASD. Previous studies have reported inconsistent results regarding the correlation between autistic traits in students and local and global information processing abnormality^18–20^. It has been indicated that the sampling may have an effect^9,77^. Future studies should employ individuals diagnosed with ASD or other general populations using the AQ to assess autistic traits to confirm the results of the present study. Third, the limitation of the present study is in psychological measures employed in it. This study explored the relationship between autistic traits and cognitive load using a task to judge face-likeness in addition to a letter-recognition task and a letter-detection task. A recent study reveals the association between autistic traits and the patterns of changes in the size of individuals’ pupils produced by cognitive styles^78^. Therefore, it is unclear whether the cognitive load from face perception was the only factor influencing the results of this study. Physiological measures, such as the size of individuals’ pupils would be beneficial in confirming whether people with higher autistic traits experience more cognitive load during local processing when face-like stimuli are present.

The results of the present study indicate that face-processing load may cause social difficulties in individuals with ASD. In many scenarios, face processing becomes essential in daily life. The present study suggests that individuals with higher autistic traits experience a greater global information processing load, and other performances, such as face identification or vocal processing, must be processed simultaneously with faces. In the clinical environment, social communication and the performance of individuals with ASD may improve if they are asked not to focus on others’ faces. To determine whether this interference is accurate, further studies should investigate the effect of face recognition on speech and language perception in more realistic situations and under dual-task conditions.

## Data Availability

The dataset generated and analyzed during the current study may be available upon reasonable request with the consent of the SFC Ethics Committee and participants.
